# Meeting at the crossroads: collaboration between information technology departments and health sciences libraries

**DOI:** 10.5195/jmla.2017.104

**Published:** 2017-01

**Authors:** Samuel King, Erica Cataldi-Roberts, Erin Wentz

## Abstract

**Objective:**

The purposes of this survey were to determine the nature and extent of collaboration between health sciences libraries and their information technology (IT) departments, to identify strengths and issues connected to this relationship, and to provide examples demonstrating exceptional collaborative success.

**Methods:**

A fourteen-question survey was sent to a broad selection of health care and academic libraries through a variety of email discussion lists and was limited to one response per institution. Convenience sampling was used to collect the responses.

**Results:**

An overwhelming majority of libraries described the relationship with their IT departments as good or excellent, and there were a variety of creative joint initiatives underway. Opportunities exist for continued and expanded library/IT collaboration.

**Conclusions:**

Good quality relationships between libraries and their IT departments are essential due to the interconnected nature of their services, and fortunately, this appears to be the norm at a variety of institutions. Mutual respect, open communication, realization of each department’s mission, and responsiveness to each other’s needs are part of what makes these relationships successful, which in turn leads to successful collaborative ventures that bode well for the future of both services.

## INTRODUCTION

The global presence of information technology (IT), increased reliance on electronic resources, fast pace of innovation, and evolution in the digital world call for increased cooperation between librarians and information technologists [1]. IT departments act as gatekeepers for many library activities, and collaboration between departments can enhance the value and visibility of libraries as well as open up new possibilities in sharing resources, reaching new patrons, and expanding services [2, 3]. This is particularly true for libraries that serve multiple hospitals, campuses, or remote departments. While reliance on electronic subscriptions has increased and electronic health records have become standard, many institutions’ IT infrastructures have not kept pace. Meeting users’ demands for easy-to-access, digital services requires cooperation between libraries and IT departments, making efficient and friendly working relationships critical.

Cowen and Edson provide a number of recommendations for effective library/IT collaboration. These include demonstrating expertise by the players in their respective areas of knowledge, focusing on process and problem solving rather than placing blame, being willing to own mistakes and the limits of one’s expertise (including the ability to give and take assistance), and committing to a collaborative and cooperative environment. When these conditions exist, common expectations and goals can be developed with the result of personal buy-in from all parties, leading to fruitful projects [4].

Previous literature, representing several types of libraries, contains other tips to promote positive progress on joint projects. Regular communication is essential [2, 5–8], and librarians and IT staff should establish relationships and get to know each other before undertaking specific projects [5, 9]. Chao and Lubas and Koelker, Bouchard, and Lutz underscore the importance of knowing and understanding the other department’s goals at the beginning of the collaboration [5, 9]. Several authors note the importance of listening skills and awareness of others’ terminology, as the meaning of words can change between different professions [7, 10, 11].

These case studies and personal experiences provide helpful insights for librarians who are seeking to develop positive relationships with their IT departments, but there has been little research to provide a more comprehensive picture. Here, the authors describe the results of a survey to characterize the depth and areas of collaboration between health sciences libraries and IT departments.

## METHODS

In collaboration with a member of our IT department, we developed a fourteen-question survey to capture the state of existing collaborations between health sciences libraries and IT departments. The supplemental appendix provides sample responses and comments from the survey. This research was declared exempt from institutional review board approval. The survey identified the size and type of the responding institutions, followed by an exploration of the nature of the relationships between their libraries and IT departments. Two questions probed the organizational relationships between the two departments. Four questions asked about the level of support that IT departments provided for typical library services. One question asked about communication methods, and the rest of the questions asked respondents to evaluate their perceptions of their relationships with the IT department and to provide examples of particularly notable collaborations.

For this study, the target population consisted of librarians and other employees at health sciences libraries in North America. During the fall of 2014, a link to the survey was disseminated to a broad selection of health care and academic libraries through the following email discussion lists: MEDLIB-L, North Atlantic Health Sciences Libraries, the Medical Library Association’s (MLA’s) Collection Development Section, MLA’s Medical Informatics Section, CANMEDLIB, and the Association of College & Research Libraries’ Information Technology Interest Group–New England Region. With permission, the invitation was also forwarded to the New York-New Jersey Chapter of MLA. Health sciences librarians were noted as the desired participants, and we requested that only one response be submitted per institution. Responses were collected using convenience sampling.

## RESULTS

Ninety-one libraries responded to the survey. The types of libraries were as follows: 33 academic (36%), 52 hospital (57%), 4 government (4%), and 2 other (2%). The number of responses for each question differed slightly, as respondents were not required to answer every question.

### Organizational structure

All respondents answered the administrative questions. Comments reflected 2 types of IT setups: institutions with a single IT department and those with an overall IT service complemented with IT specialists in the library. Twenty-six respondents shared either the same reporting line (n=16, 18%) or space (partially or totally; n=17, 19%) with their IT departments. Seven (8%) respondents shared both the same reporting line and at least a portion of their space. The remaining 65 institutions shared neither reporting lines nor spaces. One library reported to its institution’s IT department. Examples of space-sharing included computer labs and a library-based help desk. Management of computer labs varied among institutions, with IT technical support being a recurring feature. One institution commented that the transition from print journals to electronic journals opened up space that was used for IT office space.

### Information technology (IT) support for library services

#### Instruction

IT support to the 80 libraries (88% of respondents) that offer instruction came in a variety of forms, as indicated in Table 1 (subdivided by type of institution responding). Sixty (75%) of these respondents selected at least 1 form of support, but 20 (25%) of these respondents did not respond to the question about types of IT support provided.

**Table 1:** Information technology (IT) support for instruction

	Number of responses by type of institution				Total	
Type of support*	Academic	Hospital	Government	Other	n	(%)
Presentation equipment setup	23	15	1	1	40	(67%)
Support for online instruction (Blackboard, etc.)	22	2	1	0	25	(42%)
Application installation and training	20	4	0	1	25	(42%)
Instructional design support	8	0	0	0	8	(13%)
Collaborative teaching	4	0	0	0	4	(7%)
Other†	2	10	1	0	13	(22%)
Total	30	27	2	1	60	(100%)

* Respondents could select more than one option.

† Other includes responses from individuals whose comments indicate that the IT department provides no support.

Of those institutions offering instructional support, one library had a separate center for instructional design and collaborative teaching, an example of an IT-type service being offered by a library itself in its own physical environment. Various libraries identified additional IT services supporting library instruction. Of note is the use of videoconferencing support across an institution’s various locations. One IT department developed its own computer-based, instructor-led courses for providers to engage with new computer systems, one course of which was accredited through the library as the continuing medical education (CME) office. Another library’s institution had a bio-communications department that offered additional support. One more institution’s IT department provided educational support, in addition to educational technologies, to develop technical skillsets among all staff, including librarians. An instance was also reported of an IT department providing support to department-specific and institution-wide educational database development and special hospital-wide projects.

Electronic content. Eighty-three (91%) institutions responded to questions about the types of support that IT departments provide for the library’s electronic content (online books, journals, databases, catalogs, proxy servers, research guides, remote server platforms, etc.). Table 2 presents the breakdown of responses (subdivided by the type of institution responding). An additional 12 responses provided comments on these questions but did not select any of the options provided.

**Table 2:** IT support for electronic content

	Number of responses by type of institution				Total	
IT support	Academic	Hospital	Government	Other	n	(%)
IT department’s role						
Manage the entire system	2	1	0	0	3	(4%)
Provide technical support to a library-managed system	14	26	2	2	44	(55%)
Other*	15	17	1	0	33	(41%)
Total	31	44	3	2	80	(100%)
Types of support IT provides†						
Maintain the servers the content is stored on	20	22	1	1	44	(66%)
Update vendors’ IP address lists	6	13	0	0	19	(28%)
Handle the renewal and management of license terms/agreements	3	1	0	0	4	(6%)
Other support*	6*	12	1	1	20	(30%)
Total	25	39	2	1	67	(100%)

* Other and Other support include responses from individuals whose comments indicated that the IT department provided no support.

† Respondents could select more than one option.

The level of this support varied depending on the technical staff employed by the library itself and included firewall support, Internet protocol (IP) ranges, institutional network and servers (onsite or offsite, including the Office of Prevention, Education and Control [OPEC]), and so on. Other IT support included institutional intranet management, software repair and support, authentication and remote access applications, help on technical matters with the publishers of electronic journals, and issues related to cloud storage.

#### Other activities

Fifty-one (57%) out of 89 libraries reported that their IT department provided support for additional library activities, mostly centered on maintaining the institutional servers. Other types of support include storing and curating data and capture systems, maintaining the library computer labs, maintaining photocopiers, operating the wireless system, managing the online notices and policies of the library on a hospital home page, managing help desk ticketing, webcasting, and supporting library-loaned tablets and laptops. One library collaborated with IT in a video digitization project, and one hospital had a jointly developed integration of library services into its electronic medical record (EMR).

### Communication

Respondents used a variety of methods to communicate with their IT departments, as shown in Table 3 (subdivided by the type of institution responding).

**Table 3:** Communication methods between libraries and IT departments

	Number of responses by type of institution				Total	
Type of communication method*	Academic	Hospital	Government	Other	n	(%)
Face to face	30	39	2	2	73	(80%)
Phone	33	45	2	2	82	(90%)
Email	33	48	3	2	86	(95%)
Help desk (real or virtual)	27	46	3	1	77	(85%)
Hangouts, Facetime, similar programs	3	1	0	1	5	(5%)
Text messaging	2	1	1	0	4	(4%)
Social media	3	0	0	0	3	(3%)
Total	33	52	4	2	91	(100%)

* Respondents could select more than 1 option.

#### Assessment of the relationship

Seventy-five (83%) libraries identified the working relationship with their IT departments as either excellent or good, while 15 libraries (17%) identified their relationship as poor. Respondents at academic institutions and respondents in hospitals reported each option at similar rates.

When asked to comment on the strengths of the relationship, the most common response centered on the value of high-quality service and competent IT staff, with several libraries praising individual IT members. An environment of open communication, mutual respect, and skill set awareness was identified as important to good interdepartmental relations. More than one library applauded their IT departments for their understanding of the role that librarians have in the information process. Recurring themes focused on their ability to collaborate and identify or resolve problems in a collegial environment, which was deemed especially important in troubleshooting problems that cross areas of expertise. Libraries in the same reporting line as IT departments identified organizational proximity as a relationship enhancer. Serving jointly on hospital committees and sharing the same mission and commitment to the service of the patron base were also viewed as strengths. Overall, with a few exceptions, most respondents painted a bright picture of relations with their IT departments. Key descriptors for a positive relationship were “mutual support,” “trust,” and “familiarity.”

While a minority, libraries reporting a poor relationship with their IT departments identified a variety of potential causes, such as an outsourced IT service, misunderstandings based on the different focus of the two departments, the culture of the overall organization, and issues resulting from the reporting structure. The survey also asked for identification of the major challenges to a good working relationship between a library and its institution’s IT department. One key issue was the fact that each department often had different priorities and mindsets, resulting in the need to balance security issues with the end user’s need to access information, often manifesting itself through policies surrounding encryption and user authentication. Major problems occurred when there was a lack of awareness and appreciation regarding each other’s mission, expertise, and goals, which was particularly acute in a hospital environment where a library competed with clinical departments for IT services. Failure to gain support can result in the library going around its IT department, which may raise more issues. Also, funding for library-related technology issues can suffer in a clinically focused setting.

Issues of scale also impacted the IT/library relationship. Both departments provided an institution-wide service, but from the IT point-of-view, the library was just one of many departments it served. Another concern was one of physical distance. When the two departments were in locations remote from each other (especially if IT was outsourced), the resolution of any interdepartmental issues was more difficult.

Respondents stressed that better communication and knowledge of each other’s services, expertise, mission, and overall value to the institution can go a long way toward overcoming any identified challenges.

### Notable collaborative initiatives

Finally, responders were asked to share any initiatives, projects, or incidents that demonstrated a positive and creative collaborative experience between the library and IT departments.

Some institutions mentioned providing iPads in hospitals where secure access to EMRs was crucial. One library collaborated with a number of departments, including IT, to digitize and preserve osteopathic videos. Collaborations also occurred with teaching and professional development projects. One institution created a “center for teaching excellence” through a cost-sharing initiative. The center maintained a collection of classes video-recorded for future review and subsequent class design. One IT department invited the library to its exposition to present a display on research data management and open access.

Some collaborations are notable due to the personal contact afforded by individuals from the two departments. One librarian, through prior collaboration with the IT department, was brought into the design of a new intranet. This person’s role was testing new modules, implementing them in the library, and then assisting in their roll-outs to other departments. Another library had a similar experience, resulting in new codes and indexing for its LibGuides. A different library used such connections to develop an A-to-Z list for its journal collection. There were yet other examples of librarians who through established relationships worked with IT staff and vendors to agree on contracts and resolve issues using current or newly installed programs on library terminals.

## DISCUSSION

Because most respondents had distinct IT and library departments, accomplishing library goals meant that both departments needed to develop collaborative and supportive relationships. Respondents’ comments basically agreed with recommendations from the literature, noting the importance of open communication, mutual respect, and skill set awareness for good interdepartmental relationships. Thus, it follows that respondents mentioned issues stemming from a lack of these qualities when discussing challenges.

Although the results of our survey emphasized successful collaborations, respondents noted differing cultures, priorities, and needs between the two departments that made it difficult to work together smoothly, echoing themes from other studies of library/IT relations, such as a case study about moving to an open source integrated library system (ILS) by Kohn and McCloy, who noted that the two departments also had different approaches to problem-solving and planning: one department preferred concrete and the other preferred theoretical [12].

Though respondents’ comments fit with previous findings, the extent to which some of the barriers applied was greater for our survey population than for those described in other studies, most of which focused on strictly academic institutions. In general, academic library projects deal with less sensitive types of data, whereas hospital libraries face challenges due to the Health Insurance Portability and Accountability Act (HIPAA) and other regulations, especially when receiving patient-specific questions. Most comments relating to ideas of security, privacy, or federal regulations came from individuals at hospital libraries. For example, one respondent identified the “higher IT security requirements for the hospital environment” as a challenge.

Moreover, hospital systems rely on dated infrastructure designed to keep confidential information secure, which can hamper their abilities to provide access to library content. One hospital respondent identified “HUGE issues with getting programs to work through firewalls and with old browsers (which cannot be dropped because several clinical systems cannot handle updated browsers).” Most other comments about dated systems came from respondents at hospitals and reflected similar circumstances. In spite of these barriers, libraries and IT departments continue to provide relevant information services to the same client base. It is clear that collaboration and productive working relationships are needed for the future.

This study provides a baseline for library/IT cooperation in the health sciences setting and can be used as a basis for deeper research. In this survey, respondents were asked to identify the type of library they represented. Results suggest some differences between the types of institutions, such as that IT departments at academic institutions were more likely to actively support instruction and that respondents in hospitals were more likely to mention security and privacy concerns in their free-text comments. However, in-depth exploration of those differences were beyond the scope of this study and should be explored in future research. Further studies could focus on specific types of institutions or compare one or more types. Studies focusing on the differences between academic institutions and hospitals would be particularly insightful. The first consideration for such a study would be the differing service requirements based on the types of populations in each study [13]. In addition, the specific patient care needs of clinical and clinical/academic institutions versus pure academic libraries could be reviewed to identify their impact on library/IT services [14]. Another issue for future consideration is the future of independent hospital libraries, whether they will exist in the future (or will be absorbed into larger academic conglomerates), and the resulting impact of such an organizational change on library/IT collaboration [15].

Limitations to the study design and focus provide both cautions for generalizing the information and opportunities for further research. This study employed convenience sampling through online discussion lists, which tend to have low response rates and which may not be representative of the wider population. More rigorous sampling methods should be used to confirm or contradict the findings. Results only show librarians’ perspectives on their relationships with IT departments. Further studies could send the questions to IT departments to provide a more complete picture of the relationships. This study was designed to elicit information about successful collaborations, which may have biased the results toward positive responses. Like most of the existing literature, this study also used anecdotal evidence. Later studies might use experimental methods to measure the effectiveness of any particular strategy for improving relationships.

## SUPPLEMENTAL FILE

Appendix Sample survey responses and comments to specific questions
