# Supplementary material for: Meeting at the crossroads: collaboration between information technology departments and health sciences libraries
Source: J Med Libr Assoc. 2017 Jan;105(1):27–33. doi: 10.5195/jmla.2017.104 (PMC5234457; doi:10.5195/jmla.2017.104)
Supplement: Appendix [file jmla_jan17_king_app.pdf]

JMLA Journal of the Medical Library Association

SC
Supplemental content to
J Med Libr Assoc. Jan;105(1):dx.doi.org/10.5195/jmla.2017.104
www.mlanet.org
© King, Cataldi-Roberts, Wentz 2017

Meeting at the crossroads: collaboration between information technology departments and health sciences libraries

Samuel King, MLS; Erica Cataldi-Roberts, MLIS; Erin Wentz, MSLIS

APPENDIX

Sample survey responses and comments to specific questions

1. What is the size of your institution?

Answer	Responses	Percentage of responses
Under 500 persons	7	8%
501 to 1,000 persons	6	7%
1001 to 3,000 persons	27	30%
Over 3,000 persons	51	56%
Total	91	100%

2. Does your library share physical space with your information technology (IT) department.

Answer	Responses	Percentage of responses
Yes	17	19%
No	74	81%
Total	91	100%

3. Does the library offer instruction programs at your institution?

Answer	Responses	Percentage of responses
Yes	80	88%
No	11	12%
Total	91	100%

Key comments:

A. The IT department provides support for all technology within the library.
B. The IT department supports library data capture and storing activities.
C. Fixes equipment and upgrades.
D. They maintain the institution-wide network and the many workstations we have for our own and patron use.
E. Maintains student and library staff computers, printers, and copiers in the library
F. Maintains the portal on which our library's website lives.
G. IT provides support of the wireless access system.
H. Manages online content (notices, policies, etc.) of the library on the hospital's home page.
I. IT maintains webcasting services.
J. Create, train librarian on web page development, research, and archive repository.
K. IT provides a technology help desk, patron/staff computing, classroom, and web technology.
L. Efforts to digitize resources, project support, and video services.
M. IT department has provided support toward the integration of UpToDate with our EMR.

SC
Supplemental content to
J Med Libr Assoc. Jan;105(1):dx.doi.org/10.5195/jmla.2017.104
www.mlanet.org
© King, Cataldi-Roberts, Wentz 2017

4. What are the strengths in the relationship between your library and the IT department?

Key comments:

A. Good help desk, excellent campus-based support.
B. We have a good relationship, and the IT department responds quickly. Project collaboration.
C. Having an in-house IT department makes things much easier for the library.
D. Personal relationships developed over time are important. Great, smart, personable.
E. I feel like I have a fair relationship with IT because I can speak their language.
F. Open communication is important.
G. We both serve on a university-wide technology committee that meets monthly.
H. Our IT department is next to the library, and the staff there are excellent.
I. All report to the same individual (CIO) and have the same mission to the institution.
J. We know who does what in the IT department so there is a quick resolution of issues.
K. The libraries report to the CIO so there is a better chance of being a priority for IT.
L. The IT department brings the library in when metadata support is needed on a project.

5. What are the major challenges your library and IT department?

Key comments:

A. Balancing security issues with the end user's need to access information.
B. Appreciation of unique (vs. rest of institution) needs/applications of IT services to the library.
C. Different priorities—don't always align.
D. Allocating funds for up-to-date hardware and technology.
E. IT is not aware of what libraries do, what libraries provide access to.
F. Bridging the gap between ideas and what it is a feasible/realistic outcome or product.
G. Communication between departments and understanding departmental needs.
H. More involvement in the planning stages of big projects would be an improvement.

6. Are there examples of library/IT collaboration you would like to share?

Key comments:

A. Collaborative management of a shared library/computer lab space at one of the campus libraries.
B. The library IT department supports the teaching and learning mission of the library and the campus by helping to equip/support smart classrooms, smart study rooms, computer commons, and various resources/software.
C. We implemented a joint project to loan iPads. This is VERY unique in hospitals because our iPads were able to access the institution's secure network, impossible without IT support.
D. Our example is the rollout of our desktop icon to all computers within our region to our library's website.
E. The computer lab and webcasting room are both well-used, excellent collaborative projects.
F. One innovation has been a "home grown" video recording of classes, which shows both the speaking instructor and materials projected on the screen during classes.
G. Most recently, we moved from UpToDate on campus only access to UTD anywhere, thus allowing staff access to this using smartphones, etc.
H. Library and IT department has a blended service desk that provides traditional library and technology support.
I. IT recently sponsored an IT Expo highlighting the various groups within IT. They invited the library to have a table to discuss research data management and open access.
J. We developed our own A–Z list in-house, which library staff maintained supported by our IT department.

JMLA Journal of the Medical Library Association

SC

Supplemental content to
J Med Libr Assoc. Jan;105(1):dx.doi.org/10.5195/jmla.2017.104
www.mlanet.org
© King, Cataldi-Roberts, Wentz 2017

K. We collaborated in merging the home page for now two different sites. One has access to certain subscription services (databases, e-book, and journals) and the other does not.
L. We worked with IT to develop a script enabling students to access library services within Blackboard.
